# Long-term intra-fractional motion of the prostate using hydrogel spacer during Cyberknife^®^ treatment for prostate cancer – a case report

**DOI:** 10.1186/1748-717X-9-186

**Published:** 2014-08-20

**Authors:** Marcin Sumila, Andreas Mack, Uwe Schneider, Fabrizio Storelli, Jürgen Curschmann, Günther Gruber

**Affiliations:** Institute of Radiotherapy, Klinik Hirslanden, Witellikerstrasse 40, Zürich, CH-8032 Switzerland; Faculty of Science, University of Zürich, Zürich, Switzerland

**Keywords:** Prostate cancer, Stereotactic radiotherapy, Intra-fractional motion, Hydrogel spacer, Rectal toxicity

## Abstract

**Background:**

There is a trend towards hypofractionated stereotactic radiotherapy (RT) in prostate cancer to apply high single doses in a few fractions. Using the Cyberknife^®^ robotic system multiple non-coplanar fields are usually given with a treatment time of one hour or more. We planned to evaluate organ motion in this setting injecting a hydrogel spacer to protect the anterior rectal wall during treatment.

**Methods:**

A 66 years old man with low risk prostate cancer was planned for robotic hypofractionated stereotactic RT. After implantation of fiducial markers and a hydrogel spacer a total dose of 36.25 Gy in 5 fractions was given to the planning target volume (clinical target volume + 3 mm). After each beam the corresponding data reporting on the intra-fractional movement were pre-processed, the generated log-files extracted and the data analysed according to different directions: left -right (LR); anterior - posterior (AP); inferior -superior (IS). Clinical assessments were prospectively done before RT start, one week after the end of treatment as well as 1, 6 and 12 months afterwards. Symptoms were documented using Common Toxicity and Adverse Events Criteria 4.0.

**Results:**

Tolerability of marker and hydrogel implantation was excellent. A total of 284 non-coplanar fields were used per fraction. The total treatment time for all fields per fraction lasted more than 60 minutes. The detected and corrected movements over all 5 fractions were in a range of +/- 4 mm in all directions (LR: mean 0,238 – SD 0,798; AP: mean 0,450 – SD 1,690; and IS: mean 0,908 – SD 1,518). V36Gy for the rectum was 0.062 ccm. After RT, grade 1-2 intestinal toxicity and grade 1 genitourinarytoxicity occurred, but resolved completely after 10 days. On 1-, 6- and 12-months follow-up the patient was free of any symptoms with only slight decrease of erectile function (grade 1). There was a continuous PSA decline.

**Conclusions:**

Prostate movement was relatively low (+/- 4 mm) even during fraction times of more than 60 minutes. The hydrogel spacer might serve as a kind of stabilisator for the prostate, but this should be analysed in a larger cohort of patients.

## Introduction

Prostate cancer is the most common non cutaneous cancer type in men. However the best treatment strategy is a matter of controversy. There is agreement regarding equivalency of radical prostatectomy and radiotherapy (RT) especially in low risk cancer. A recent publication including more than 50000 patients could even find a superiority of RT in compared to radical prostatectomy in all defined risk groups [1].

The goal of treatment is tumour control and maintaining quality of life. Hypo-fractionated RT with robotic radiosurgery is an attractive method with growing evidence of efficacy [2–6], pooled analysis in [6]. The therapeutic goal is the application of a high radiation dose to the prostate and to spare the surrounding healthy tissue at the same time. To reach this prerequisite it is important to take into account organ motion of the prostate itself but also of organs at risk like e.g. the bladder and the rectum. This is especially true for hypofractionated stereotactic RT, where high doses per fraction and therefore small safety margins around the prostate are applied. The implantation of gold markers into the prostate has been widely adopted, and with these fiducials the actual prostate position can be monitored and tracked before and even during every fraction.

A relatively new development is a hydrogel spacer, which can be injected between prostate and rectum. By increasing the distance the anterior rectal wall can be better spared from the high dose region of the irradiation [7–10]. One can hypothesize, that the hydrogel spacer may stabilize the prostate and reduce its motion. We report here our experience with the first patient treated on the Cyberknife^®^ robotic RT system using both, implanted fiducial markers and hydrogel spacer.

## Patient and methods

A 66 years old patient with localized low risk prostate cancer (T2a, Gleason-Score 6, PSA 5 ng/ml) was referred to our department for stereotactic irradiation. After informed consent fiducial markers (Heider Medical Products, Daeniken, Switzerland) were implanted into the prostate by the urologist. At the same procedure the spacer gel (SpaceOAR^®^ System, Augmenix Inc., Waltham, MA) was injected transperineal under ultrasound guidance into the space between the prostate and the rectum. Details about the procedure are given elsewhere [11]. 7 days after the implantation the planning computer tomography was done, transferred to the MultiPlan^®^ inverse planning system and fused with post interventional magnetic resonance imaging allowing an optimal visualization of intra-pelvic organs, gold markers and the hydrogel. Following structures were contoured: Prostate, seminal vesicles, rectum, bladder, penile bulb, urethra, bowel and hydrogel. The clinical target volume (CTV) included the prostate and the base of seminal vesicles.

**Figure 1:**
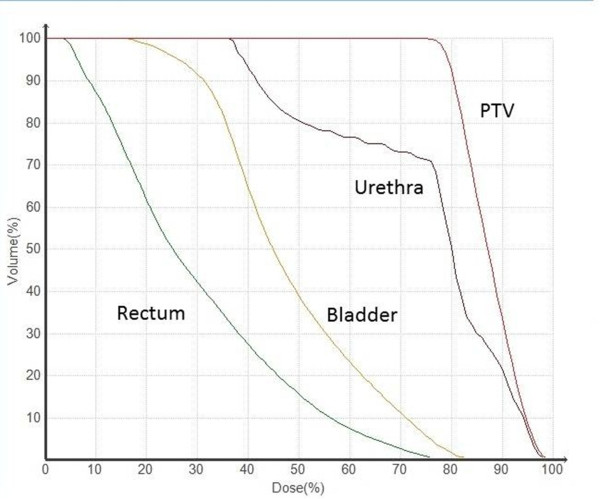
**Dose-volume histogram (DVH) for PTV, rectum, bladder and urethra.**

The planning was done according to the so-called Seattle protocol. For the planning target volume (PTV) the CTV was expended with a 3 mm margin in all directions and a total dose of 36.25 Gray was delivered to the PTV in 5 fractions given every second day. The dose-constraint for the rectum proposed in the protocol is V36Gy <1 ccm.A total of 284 non-coplanar fields were used per fraction. The treatment time for all fields per fraction lasted from 59 to 68 minutes.The dose-volume histogram is shown in Figure 1.

During each fraction the corrections which are detected by the x-ray system of the Cyberknife^®^ are monitored and compensated by the robotic manipulator. The time interval between the positioning control images were 15 seconds at the beginning and extended up to 45 seconds during each fraction.

After each beam the corresponding data reporting on the intra-fractional movement were pre-processed, the generated log-files extracted and the data analysed according to different directions (LR, AP, IS).

Clinical assessments were prospectively done before RT start, one week after the end of treatment as well as 1, 6 and 12 months afterwards. Symptoms were documented using Common Toxicity and Adverse Events Criteria (CTCAE 4.0).

## Results

The patient tolerated the spacer and marker implantation very well. No pain, no rectal discomfort or other symptoms related to the procedure or to the spacer itself were noted after application or during follow-up. According to the planning system V36Gy for the rectum was 0.062ccm.

**Figure 2:**
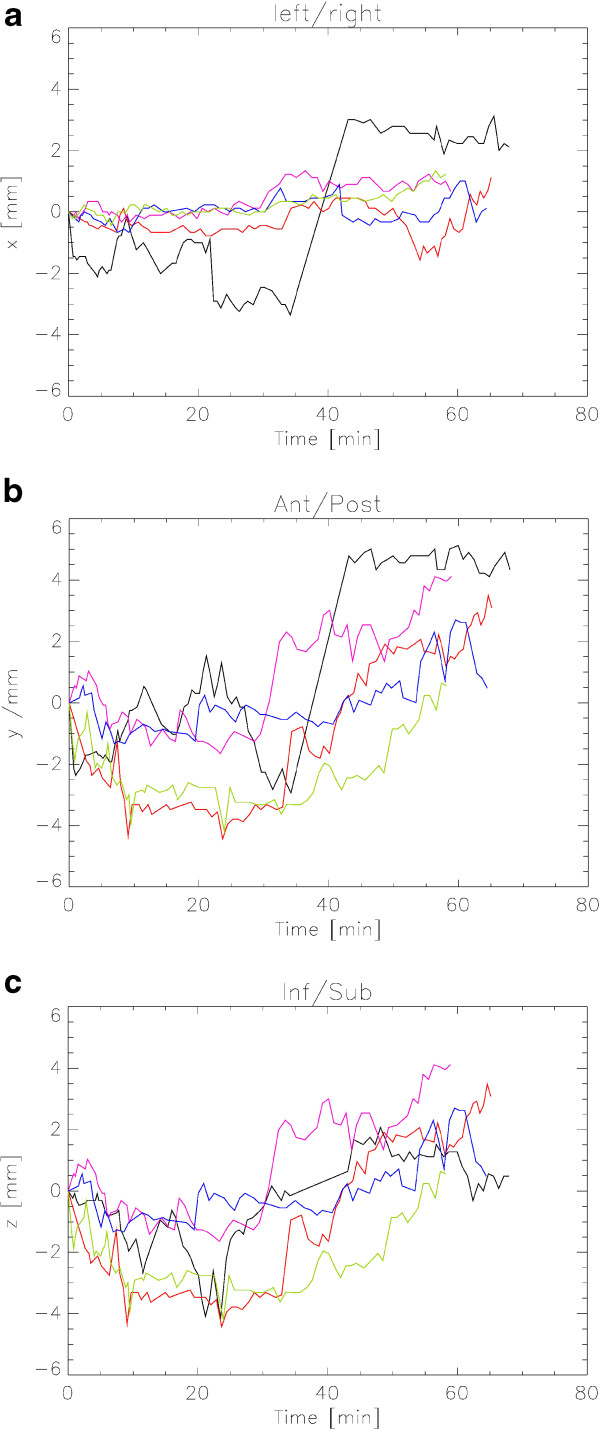
**Plot of the prostate movement during one course of Cyberknife** ^®^ **treatment in the left-right- (a), anterior-posterior- (b) and inferior-superior-direction (c).** The movement was measured using two orthogonal x-ray projections and the implanted gold markers. The different colours indicate the different fractions (F): 1st F black, 2nd F red, 3rd F magenta, 4th F blue, 5th F green. It should be noted that during the actual treatment the position of the treatment table was adjusted after each measurement.

Before treatment pelvic functions were normal without any genitourinary or gastrointestinal problems. Few days after the completion of RT grade 2 proctitis and grade 2 diarrhoea with grade 1 faecal incontinence and grade 1 rectal hemorrhage occurred. In addition, there were grade 1 urinary urgency, grade 1 urinary frequency and grade 1 cystitis noninfectiva. All these symptoms disappeared completely after ten days. During this time period the patient used only Scheriproct^®^ suppositories (prednisolon and cinchocain) but no other drugs. On 1-, 6- and 12-months follow-up he was free of any symptoms with only slight decrease of erectile function (grade 1). We observed very good PSA-response with 0.95 ng//ml, 0.57 ng/ml respective 0.34 ng/ml at 3, 6 respective 12 months after RT.The evaluation of the log-files showed following results: The detected and corrected movements over all 5 fractions are in a range of +/- 4 mm in all orientations (LR: mean 0,238 – SD 0,798; AP: mean 0,450 – SD 1,690; and IS: mean 0,908 – SD 1,518). Details of the prostate movements over time are given in Figure 2a-c.

## Discussion

High dose conformality and steep dose gradients are particularly important when treating the prostate due to the close proximity of dose-limiting structures, such as the rectum and bladder. This is even more important, when high-dose single fractions are given, like in radiosurgery or stereotactic fractionated RT. For that purpose the Cyberknife^®^ system is used in our institution. The robotic mobility enables the delivery of a large number of non-isocentric, non coplanar beams individually directed at unique points within the intended target. Nevertheless, knowing that intra-fraction prostate movement is at random and unpredictable [12], the safely and accurately delivering of irradiation to the prostate represents a challenge for any external beam radiation delivery system. Especially differences in rectal and bladder filling during daily RT sessions can result in significant prostate motion. In a review it was argued that prostate movement is greatest in the AP and IS directions [13, 14]. The standard deviations varied from 1.5 to 4.1 mm in the AP, from 0.7 mm to 1.9 mm in the LR and from 1.7 mm to 4.5 mm in the IS direction. Our measurements are comparable and they are lying in the lower range of the above mentioned data. It has been also shown, that over the course of 8 to 16 minutes prostate movement can be as much as 9.1 mm AP, 8.6 mm IS and 4.8 mm LR [15]. Therefore, prostate motion could be expected to be even higher during the quite long treatment sessions of more than one hour at the Cyberknife^®^. We have observed movements over all 5 fractions to be in the range of +/- 4 mm. One might hypothesize that the spacer-gel has some possibility of stabilization of the prostate.

Conventional image-guided RT technologies provide image guidance for the pre-treatment setup and can be used during treatment delivery to detect intra-fraction organ motion. However treatment accuracy is only guaranteed when the information acquired from the image guidance system is used automatically to correct the beam delivery in real time. Due to the close proximity and the fact that a safety margin of normally 3 to 5 mm is given to counteract patient set-up inaccuracies and organ motion as discussed above it is nearly impossible to spare e.g. the anterior rectal wall from the high dose region. According to our preliminary experience it seems appropriate to use safety margins of 4 mm without corrections even in long lasting treatment fractions, if a hydrogel spacer is used. As the Cyberknife^®^ is correcting the prostate motion in real-time, it can be discussed, if a safety margin is necessary at all. On the other hand one cannot exclude small movements during two measuring points or even during one single beam. Furthermore, rectal side effects were mild and transient as discussed below. Therefore we keep these 3 mm for PTV definition as proposed in the Seattle protocol.

The injection of a spacer seems logical to enlarge the distance between the target volume (prostate) and the organ-at-risk (anterior rectal wall). Despite its increased use clinical data are still rare. Two prospective studies are published with a median follow-up of 12 weeks and 12 months, respectively [8, 10]. Furthermore, the total number of patients is quite low (10 and 52 patients, respectively). So far, acute and chronic side effects are mild, most likely due to a decrease of the high-dose region in the surrounding organs, especially the rectum. There were no grade 3-4 toxicities. Gastrointestinal (GI) grade 1 acute side effects were reported in 37% [10] and 50% [8], grade 2 in 0% and 12%, respectively. Data regarding late toxicity were documented only in one publication [10]: grade 1 GI toxicity occured in 4%. There was no late GI toxicity higher than grade 1.

Despite spacer our patient suffered from grade 1 and 2 toxicities, but symptoms resolved within 10 days and the patient was without any symptoms at last follow-up one year after treatment. This is in concordance with the published data showing some worsening of bowel and urinary function within the first 3 months, which have been resolved within half a year and remained so beyond 5 years of follow-up [6, 16]. V36Gy for the rectum in our patient was 0.062 ccm, far below the given dose constraint of 1 cm^3^.

Several studies [17–21] have evaluated the efficacy and side effects and of fractionated stereotactic RT using the robotic system. An overview is given in Table 1.

**Table 1:** **Cyberknife** ^®^ **publications for primary treatment of prostate cancer**

Author	Dose scheme	No. of patients	Median follow-up (moths)	FFBF (in % by risk group)			Early toxicity in %						Late toxicity in %		Patients receiving ADT in %
				low (l)	intermediate (i)	high (h)	GU			GI			GU	GI	
							G1	G2	G3	G1	G2	G3	G3	G3	
Friedland et al. 2009 [5]	7 and 7.25 Gy × 5	112	24		l,i,h: 97.3								0	1	19
Bolzicco et al. 2010 [17]	7 Gy × 5	45	20	l,i:100			36	11	0	24	24	0	22	0	38
Freeman et al. 2011 [18]	7 and 7.25 Gy × 5	41	60	92.7						*			25	0	0
Kang et al. 2011 [19]	8,8.5, and 9 Gy × 4	44	40	100	100	90.8	n.A.	14	0	n.A.	9	0	0	0	87
King et al. 2012 [20]	7.25 Gy × 5	67	32	94 (4-year)									3	0	0
McBride et al. 2012 [21]	7.25 and 7.25 Gy × 5	45	44	97.7 (3-year)			59	19	0	31	7	0	2	5	0
Oliai et al. 2012 [2]	7,7.25 and 7.5 Gy × 5	70	31	100	94.7	77.1	56	19	4	17	4	0	3	0	33
Katz et al. 2013 [4]	7 and 7.25 Gy × 5	304	60	97.7 (3-year)	90.7	74.1	72-75	4-5	0	75-76	4	0	2	0	19
Chen et al. 2013 [3]	7 and 7.25 Gy × 5	100	28	100	100	88	36	35	0	35	5	0	0	<1	11
King 2013 [6], pooled date	7-7.25 Gy × 5	1100	36	95	83	78									14

*Acute symptoms typically resolved within one month of treatment completation.

FFBF: freedom from biochemical failure, GU: genitourinary, GI: gastrointestinal, G: Grade, ADT: androgen deprivation therapy.

To conclude this is the first evaluation of prostate movement using a hydrogel spacer for Cyberknife^®^ treatment with 5 fractions lasting more than 60 minutes each. According to our measurements a CTV to PTV safety margin of 4 mm would be sufficient to cover all prostate movements even without any correction. This might have implications in complex and perhaps long-lasting gantry-based linac treatments for prostate cancer. The hydrogel spacer might serve as a kind of stabilisator for the prostate, but this should be analysed in a larger cohort of patients.

## Abbreviations

ADT: Androgen deprivation therapy
AP: Anterior – posterior
CTV: Clinical target volume
FFBF: Freedom from biochemical failure
G: Grade
GI: Gastrointestinal
GU: genitourinary
IS: Inferior –superior
LR: Left – right
PTV: Planning target volume.
